# BoLA-DRB3 gene polymorphism and FMD resistance or susceptibility in Wanbei cattle

**DOI:** 10.1007/s11033-012-1793-7

**Published:** 2012-06-29

**Authors:** Wei Lei, Qinglong Liang, Luo Jing, Chengmin Wang, Xiaobing Wu, Hongxuan He

**Affiliations:** 1 Key Laboratory of Animal Ecology and Conservation Biology, Institute of Zoology, National Research Center For Wildlife-Borne Diseases, Chinese Academy of Sciences, Beijing, 100101 People’s Republic of China; 2 Key Laboratory of Animal Genetics, Faculty of Animal Science, Suzhou Vocational and Technical College, Suzhou, 234000 Anhui People’s Republic of China; 3 Anhui Provincial Key Laboratory of the Conservation and Exploitation of Biological Resources, College of Life Sciences, Anhui Normal University, Wuhu, 241000 People’s Republic of China

**Keywords:** BoLA-DRB3, Polymorphism, FMD, Resistance, Susceptibility

## Abstract

For the further characterization of foot-and-mouth disease virus (FMDV)-induced foot-and-mouth disease, we investigated the association between polymorphism of BoLA-DRB3 gene and FMD resistance/susceptibility of Wanbei cattle challenged with FMDV. One hundred cattle were challenged with FMDV and exon 2 of BoLA-DRB3 genes was amplified by hemi-nested polymerase chain reaction from asymptomatic animals and from animals with FMD. PCR products were characterized by the RFLP technique using restriction enzymes Hae III. The results revealed extensive polymorphisms, 6 RFLP patterns were identified. By analyzing alleles and genotypic frequencies between healthy and infection with FMD cattle, we found that allele Hae III A was associated with susceptibility to FMD in Wanbei cattle (*P* < 0.05), whereas Hae III C was associated with resistance to FMD (*P* < 0.01) and may have a strong protective effect against FMD. Hae IIICC and Hae III BC genotype were associated with resistance to FMD (*P* < 0.01). By contrast, Hae III AA genotype was associated with susceptibility to FMD (*P* < 0.01). Sequence analysis show that 89 amino acids were translated in exon 2 of BoLA-DRB3 and 13.70 % of nucleotide mutated, which resulted in 14.61 % of amino acid change. One PKC, one Tyr and one CAMP phosphorylation were increased; the hydrophobicity and secondary structure of proteins produced change after amino acid substitution. These results revealed that Wanbei cattle had the ability of resistance to disease by mutation which result changes of the protein structure to perform the regulation of the cell using different signaling pathways in the long process of choice evolution.

## Introduction

The foot-and-mouth disease virus (FMDV) was a member of the genus *Aphthovirus* in the family Picornaviridae. There were seven immunologically distinct serotypes—O, A, C, SAT 1, SAT 2, SAT 3 and Asia 1—and over 60 strains within these serotypes. FMDV serotypes and strains vary within each geographic region. Serotype O was the most common serotype worldwide. FMDV can infect most or all members of the order Artiodactyla (cloven-hooved mammals), as well as a few species in other orders. On most continents, cattle were usually the most important maintenance hosts for FMDV. Although this epizootic can be controlled by the use of a chemically inactivated whole virus vaccine, many countries have abandoned vaccination due to several disadvantages of this vaccine [1].

Evolutionary biology was increasingly concerned with the study of infectious diseases [2–4], for example host heterogeneity in disease susceptibility, which was thought to play a major role in disease persistence [5–7]. Immunogenetic studies and molecular advances provide powerful tools for understanding protective and pathogenic mechanisms in such infectious disease and make possible the study of the genetic basis of host resistance [8–11]. Specifically, in the case of disease infection, genes located in different regions of the host genome have been implicated in resistance to infection. Immunogenetics has mainly focused on major histocompatibility complex (MHC). MHC was genetic region consisting of a group of closely linked and highly polymorphic loci on chromosome, which played a central role in the immune response and immunological recognition. MHC was unique in their general importance for conferring susceptibility or resistance to infectious and autoimmune diseases [12–14].

Many studies showed that polymorphism in the genes for BoLA class II molecules determines the specificity of the immune response and plays a role in conferring resistance or susceptibility to: (a) chronic autoimmune disorders such as rheumatoid arthritis, insulin-dependent diabetes mellitus, pemphigus vulgaris, and multiple sclerosis [15]; (b) infectious diseases such as tuberculoid leprosy and malaria [16, 17]; and (c) malignancies such as carcinoma and melanoma [18–21]. Among *BoLA* class II genes, BoLA-DRB3 functional genes, which were highly polymorphic, were found to have a stronger association with resistance/susceptibility to bovine leukemia virus [22], dermatophilosis and mastitis [23] than DRB2 or DQB. The range of MHC class II BoLA-DRB3 gene polymorphisms associated with resistance and susceptibility to some infection disease in cattle was confirmed [22, 24].

Up to now, study for BoLA-DRB3 gene polymorphisms associated with resistance or susceptibility to FMD has not been reported in cattle. There was a lack of comparative data from resistant and susceptible cattle to clarify differences in immunological processes, notably molecular levels. We thus decided to investigate BoLA-DRB3 as a candidate gene for FMD resistance in Wanbei cattle. The objective of this preliminary study was to describe the genetic variability and allele frequency in the exon 2 of BoLA-DRB3 genes using PCR-RFLP method and to investigate BoLA-DRB3 alleles association with resistance and susceptibility to FMD in Wanbei cattle, to set a direction for the future studies.

## Materials and methods

### Cattle sampling and experimental FMDV challenge

Wanbei cattle of 1–2 years of age were collected from Anhui, Jiangsu and Henan province in China. In order to contrast FMD-susceptible and FMD-resistant individuals, cattle were experimentally challenged with FMDV. First, the FMD serology status of each animal was established using FMDV-specific antibodies assay, and only seronegative individuals were included in these experiments. 100 cattle sampling were brought to the experimental area isolated for experimental challenge (at least 3 weeks of quarantine). Subsequently, cattle were inoculated with 5.0 ml of blood from FMDV infected cattle. Inoculated cattle were monitored for 3 weeks. Then we classified the cattle into two types: (a) FMDV infected but clinically normal cattle; and (b) cattle with FMD. Two approaches were carried out. First, we used animals experimentally challenged with FMDV, and we compared the BoLA-DRB3 genotypes of susceptible (cattle with FMD) and resistant (FMDV infected but clinically normal cattle. Second, we compared BoLA-DRB3 allelic frequencies in cattle infected by FMDV but clinically normal and cattle with FMD.

### DNA extraction and amplification of BoLA-DRB3

100 blood samples of Wanbei cattle, which included 68 healthy and 32 infected with FMD. Genomic DNA was extracted from whole blood (100 μl) by the phenol–chloroform extraction method described by Sambrook with some modifications. The concentration and purity of obtained DNA were assessed by spectrophotometery and electrophoresis in 1 % agarose gels, respectively. Exon 2 of BoLA-DRB3 gene was amplified by hemi-nested PCR, described by Miretti, to improve the specificity of the PCR product. Primers (FHL010: 5′-ATCCTCTCTCTGCAGCACATTTCC-3′; RHL011: 5′-CTTGAATTCGCGCTCACCTCGCCGCTG-3′; RHL012: 5′-TCGCCGCTGCACAGTGAAACTCTC-3′), described by Van Eijk, were used in the PCR reaction. Briefly, the first stage PCR was performed in a final volume of 20 μl containing 50 ng of template DNA, 0.5 pm of primer FHL010 and RHL011, 2 μl PCR buffer, 1.75 mM MgCl_2_, 0.25 mM dNTPs, 1.5 U Taq DNA Polymerase (Shanghai Sangon). This reaction system was predenatured at 94 °C for 4 min followed by 12 cycles of denaturizing (94 °C for 1 min), annealing (60 °C for 1 min) and elongation (72 °C for 1 min) and a final extension at 72 °C for 5 min. 2 μl of the first stage PCR product was used as template DNA. For the second stage PCR in a final volume of 40 μl containing 0.5 pM of primer FHL010 and RHL-012, 4 μl PCR buffer 1.75 mM MgCl_2_, 0.25 mM dNTPs and 2 U Taq DNA polymerase. The solution was predenatured at 94 °C for 4 min followed by 30 cycles of denaturizing (94 °C for 60 s), annealing (63 °C for 45 s), and elongation (72 °C for 45 s) and a final extension (72 °C for 5 min). Then, 5 μl of PCR products were subjected to electrophoresis in a 2 % agarose gel in order to check the quality and specificity of amplified DNA fragment.

### Restriction fragment length polymorphism (RFLP) analysis

Discrimination of polymorphism in *BoLA* class II *DRB3* gene in this paper was performed by RFLP of the amplified fragments utilizing restriction sites unique for HaeIII. Twenty microliter of PCR product that contained a fragment of the expected size was then digested with 10 Units of each restriction enzyme used in this study in final reaction on volume 25 μl. The reaction mixture was incubated at 37 °C for HaeIII in water bath over night. Restriction fragments were revealed by gel electrophoresis on 12 % PAGE under 150 V for 5 h, using Msp I digested pBR322/MspIas a molecular marker.

### Statistic analyses

The allele frequency (P) and genotypic frequency (G) were calculated based on Wang [25]. Two approaches were carried out. First, we compared the BoLA-DRB3 genotypes of susceptible (infection) and resistant (healthy) cattle. Second, we described the genetic variability and compared BoLA-DRB3 allelic frequencies between healthy and infection with FMD cattle. The statistical significance of differences between genotype and resistance/susceptibility to FMD were analyzed using SPSS 18.0 software, the difference is considered statistically significant when *P* was 0.05.

## Results

### PCR products

**Fig. 1:**
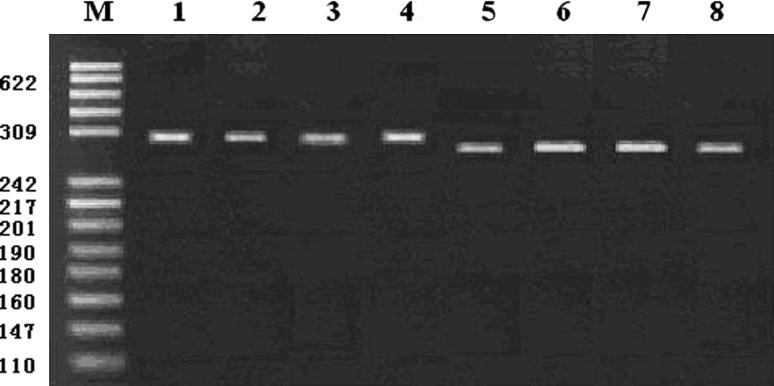
PCR products are examined by 2 % agarose gel electrophoresis

PCR products were detected by 2 % agarose gel electrophoresis, the results showed that the amplified products were 302 and 284 bp in length with good specificity, which consistent with the expected fragment (Fig. 1).

### Identification of restriction patterns

**Fig. 2:**
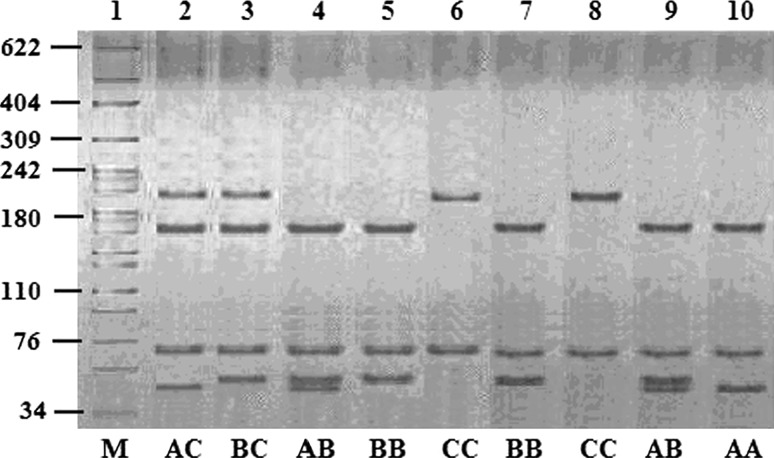
Bind patterns of DRB3 locus digested with Hae III

The 284 bp fragment of the BoLA-DRB3.2 gene in this study were digested with Hae III. 6 RFLP patterns were identified (167/65/48/4, 167/65/52, 219/65, 167/65/52/48/4, 219/65/48/4, 219/65/52 bp) and named AA, BB, CC, AB, AC and BC. 3 allele were found in Wanbei cattle and named A, B and C. The results were showed in Fig. 2.

### Analysis of DNA sequences

**Fig. 3:**
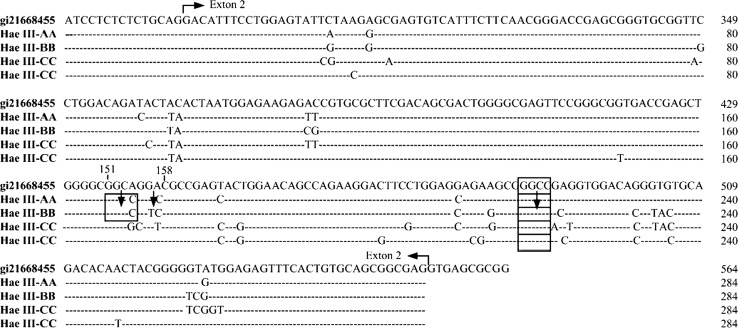
The blast of sequence of DRB3 with different genotype

Cloning and sequencing results showed that 284 bp fragments were amplified, including 15 bp intron 1 and 269 bp exon 2. 38 bases mutations were found by aligning the sequence of homozygous and GenBank (gi:21668455) (Fig. 3), and mutations produced HaeIII polymorphism at 154, 155, 156 and 157 position (Table 1). 89 amino acids were translated in exon 2 of BoLA-DRB3 (http://www.ncbi.nlm.nih.gov/BLAST/Blast.cgi). Among these amino acid sequences, there were 13 substitutions by comparing with the GenBank sequence (NP-001012698). One PKC, one Tyr and one CAMP phosphorylation were increased after amino acid substitution (http://www.expasy.org/prosite/). However, one α-helix structure was reduced in the secondary structure of protein (http://npsa-pbil.ibcp.fr/); protein hydrophobic was significantly lower from 40 to 50 (http://www.expasy.org/tools/protscale.html).

**Table 1:** Variant position and sequence of three RFLPs-HaeIII of exon 2

Genotypes	Partial sequence (5′ → 3′)	Variant position	Variant sequence	Number of digestion point	Fragment size (bp)
AA	151-GGCCGGCC-158			3	167, 65, 48, 4
BB	151-GGCCGTCC-158	156	G → T	2	167, 65, 52,
CC	151-GGCGCGTC-158	154	C → G	1	219, 65
		155	G → C		
		157	C → T		
	151-GGCAGGAC-158	154	C → A		
		157	C → A		

### The frequency of BoLA-DRB3 alleles in infected FMD and healthy cattle

There were the same alleles and genotype in healthy and infection with FMD cattle. The numbers and frequency of the BoLA-DRB3 alleles in infected FMD and healthy Wanbei cattle were shown in Table 2. The frequency of HaeIIIA, HaeIIIB and HaeIIIC were 0.2059, 0.3309 and 0.4632 in healthy cattle respectively. The frequency of HaeIIIA, HaeIIIB and HaeIIIC were 0.4844, 0.1875 and 0.0469 in FMD cattle, respectively. The frequency of HaeIIIAA, HaeIIIBB, HaeIIICC, HaeIIIAB, HaeIIIAC and HaeIIIBC were 0.0882, 0.1324, 0.2648, 0.1176, 0.1176 and 0.2794 in healthy cattle, respectively; the frequency of HaeIIIAA, HaeIIIBB, HaeIIICC, HaeIIIAB, HaeIIIAC and HaeIIIBC were 0.2344, 0.1875, 0.0625, 0.1563, 0.1250 and 0.0625 in FMD cattle, respectively.

**Table 2:** Allele frequencies of BoLA-DRB3 for FMDV-challenged cattle

	Health (*n* = 68)			FMD (*n* = 32)	
Allele	No	Frequency	Allele	No	Frequency
HaeIII A	28	0.2059	HaeIII A	35	0.4844*
HaeIII B	45	0.3309	HaeIII B	19	0.1875
HaeIII C	63	0.4632**	HaeIII C	3	0.0469

*Note* The same alleles between positive and negative FMD

* P < 0.05, ** P < 0.01

## Discussion

### The BoLA-DRB3 polymorphism in Wanbei cattle

Statistical results showed that 38 bases mutations were found and polymorphism sites at positions 154, 155, 156 and 157 in Wanbei cattle. Sequence analysis showed that 89 amino acids were translated in exon 2 of BoLA-DRB3 and 13.70 % of nucleotide mutated, which resulted in 14.61 % of amino acid change. One PKC, one Tyr and one CAMP phosphorylation were increased and the hydrophobicity and secondary structure of proteins produced changes after amino acid substitution. The results in this study were consistent with findings obtained by Wang [25]. These results revealed that cattle had the ability of resistance to disease by mutation which result changes of the protein structure to perform the regulation of the cell using different signaling pathways in the long process of choice evolution [25].

### BoLA-DRB3 alleles associated with resistance and susceptibility to FMD

There were the same alleles and genotype in healthy and infection with FMD cattle. The numbers and frequency of the different *BoLA*-*DRB3* alleles in infected FMD and healthy Wanbei cattle were shown in Table 2. HaeIII C allele was present at a significantly higher frequency in healthy cattle as compared with cattle with FMD (healthy 0.4632; FMD 0.0469). The frequency of cattle infected FMD for HaeIII A (0.4844) was higher than healthy cattle for the same allele (0.2059). No other alleles were associated with a statistically significant difference between healthy and infection with FMD cattle. This result suggested that allele HaeIII A was associated with susceptibility to FMD in Wanbei cattle (*P* < 0.05), whereas HaeIII C was associated with resistance to FMD (*P* < 0.01) and may have a strong protective effect against FMD.

We compared frequencies of *BoLA*-*DRB3* genotypes in healthy and infection with FMD cattle (Table 3). The frequency of HaeIII CC and HaeIII BC was higher in healthy cattle than in FMD cattle for the same genotypes (healthy 0.2648, 0.2794; FMD 0.0625, 0.0625). This result suggested that HaeIII CC and HaeIII BC genotype were actually associated with resistance to FMD (*P* < 0.01). By contrast, HaeIII AA genotype was associated with susceptibility to FMD (*P* < 0.05). In the case of Hae III BB, Hae III AB and HaeIII AC genotypes, the differences between healthy and FMD cattle were not significant.

**Table 3:** Genotypic frequencies of BoLA-DRB3 for FMDV-challenged cattle

	Health (*n* = 68)			FMD (*n* = 32)	
Genotype	No	Frequency	Genotype	No	Frequency
BstYI AA	6	0.0882	BstYI AA	15	0.2344**
BstYI BB	9	0.1324	BstYI BB	6	0.1875
HaeIII CC	18	0.2648**	HaeIII AA	2	0.0625
HaeIII AB	8	0.1176	HaeIII AB	5	0.1563
HaeIII AC	8	0.1176	HaeIII BB	4	0.1250
HaeIII BC	19	0.2794**	HaeIII AD	2	0.0625

*Note* The genotypes between positive and negative FMD

* P < 0.05, ** * P* < 0.01

BoLA-DRB3 played a key role in immune response to foot-and-mouth disease [26]. Baxtera et al. [27] studied the associations between bovine MHC DRB3 alleles and their binding pockets with the immune response to a 40-mer peptide derived from FMDV VP1. Eighteen different DRB3 alleles were detected in a crossbred (Charolais and Holstein) cattle population, with several exhibiting highly significant associations with antibody response. Allele DRB3*1601 was correlated with relatively low IgG1 and IgG2 responses (*P* < 0.001), whereas DRB3*1001 was associated with relatively high IgG1 and IgG2 responses (*P* < 0.001). The data indicated that the DRB3 alleles were critical for determining the degree of immune response. In this paper, Hemi-nested PCR-RFLP method was used for identification the frequency of *BoLA*-*DRB3* alleles and genotypes in Wanbei cattle. 6 RFLP patterns were found with enzymes Hae III. Based on analysis of significant difference of allele and genotypic frequencies in infected FMD and healthy cattle, our results demonstrated the existence of alleles associated with resistance and susceptibility to FMD. The allele Hae III A was associated with susceptibility in Wanbei cattle (*P* < 0.05); But the allele Hae III C was associated with resistance (*P* < 0.01); the genotypes of Hae IIICC and HaeIIIBC were associated with resistance to FMD (*P* < 0.01). By contrast, Hae IIIAA genotype was associated with susceptibility to FMD (*P* < 0.05).

The highly polymorphic bovine MHC (BoLA)-DRB gene had been implicated in the resistance and susceptibility to a broad range of diseases [13]. Earlier studies showed that some bovine MHC class II BoLA-DRB3.2 gene polymorphic were correlated with resistance and susceptibility to the development of persistent lymphocytosis (PL) cased by bovine leukaemia virus (BLV) infection [22, 28]. Subsequently, Panei et al. [29] identified 17 BoLA-DRB3 alleles defined according to the PCR-RFLP nomenclature and represented the distribution of the allele frequent in Holando-Argentino dairy cattle. Alleles *BoLA*-DRB3.2*11, *23 and *28 mediating resistance to PL and alleles *BoLA*-DRB3.2*22 and *24 mediating susceptibility to PL were observed, alleles *BoLA*-DRB3.2*25 and *40 also showed signification association to PL. The DRB3 polymorphism has also been observed to be associated with resistance or susceptibility to dermatophilosis, cystic ovarian and mastitis [30–33].

MHC molecules were important in disease resistance for many infectious agents. This study was the first to investigate the association between BoLA-DRB3 polymorphism and FMD among Wanbei cattle natural population and demonstration that MHC can have a role in the clearance of FMDV infections. This study suggested that MHC-mediated immune recognition can be an important variable in susceptibility to FMDV infections. However, the limitation of this preliminary study was that the sample size was not relatively large (*n* = 100) and these finding may not apply to all Wanbei cattle. Additional studies were required to define in detail the mechanism of the association between susceptibility to FMD and polymorphism of MHC class II alleles. Therefore, alterations in the biochemical nature and physiological function of BoLA-DRB3 molecules in association with resistance or susceptibility to FMD were also worthy of further examination.
